# Neuropathology determines whether brain systems segregation benefits cognitive performance

**DOI:** 10.1162/IMAG.a.138

**Published:** 2025-09-09

**Authors:** Annabell Coors, Weiyi Zeng, Ulrich Ettinger, Monique M.B. Breteler

**Affiliations:** Population Health Sciences, German Center for Neurodegenerative Diseases (DZNE), Bonn, Germany; Cognitive Neuroscience Division, Department of Neurology, Columbia University Vagelos College of Physicians and Surgeons, New York, NY, United States; Department of Psychiatry, Psychotherapy and Psychosomatic Medicine, University Medical Center Halle, Halle (Saale), Germany; Department of Psychology, University of Bonn, Bonn, Germany; Institute for Medical Biometry, Informatics and Epidemiology (IMBIE), Faculty of Medicine, University of Bonn, Bonn, Germany

**Keywords:** brain systems segregation, resting-state fMRI, cognition, neuroaxonal damage, aging

## Abstract

The human brain is a large-scale network, containing multiple segregated, functionally specialized systems. With increasing age, these systems become less segregated, but the reasons and consequences of this age-related reorganization are largely unknown. Thus, after characterizing age- and sex-specific differences in the segregation of global, sensorimotor, and association systems using resting-state functional MRI data, we analyzed how segregation relates to cognitive performance in both classical and eye movement tasks across age strata and whether this is influenced by the degree of neuropathology. Our analyses included 6,455 participants (30–95 years) of the community-based Rhineland Study. System segregation indices were based on functional connectivity within and between 12 brain systems. We assessed cognitive performance with tests for memory, processing speed, executive function, and crystallized intelligence and oculomotor tasks. Multivariable regression models confirmed that brain systems become less segregated with age (e.g., global segregation: standardized regression coefficient (ß) = -0.298; 95% confidence interval [-0.299, -0.297], p < 0.001) and that in older age this effect is stronger in women compared to men. Higher segregation benefited memory (especially in young individuals) and processing speed in individuals with mild neuropathology (not significant after multiple testing correction). *Lower* segregation benefited crystallized intelligence in 46- to 55-year-olds. Associations between segregation indices and cognition were generally weak (ß ~ 0.01–0.06). This suggests that optimal brain organization may depend on the degree of brain pathology. Age-related brain reorganization could serve as a compensatory mechanism and partly explain improvements in crystallized intelligence and the decline in fluid cognitive domains from adolescence to (late) adulthood.

## 1 Introduction

The human brain is a large-scale network, which can be divided into multiple interacting brain systems (Chan et al., 2014). These systems can be assigned to two categories: the sensorimotor systems and the association systems. Association systems hereby contain those that are mainly responsible for integrating information from a wide range of tasks and across multiple modalities, whereas sensorimotor systems are those that mainly process incoming sensory input and produce motor output (Chan et al., 2014; Wig, 2017). The human aging process is associated with decreased specialization in brain function (Goh, 2011). Studies of functional connectivity patterns across the healthy adult lifespan have revealed that increasing age is associated with decreasing connectivity within systems and increasing connectivity between systems (so-called age-related dedifferentiation of neural function) (Betzel et al., 2014; Chan et al., 2014; Song et al., 2014). Furthermore, systems have been found to show different trajectories in their dedifferentiation patterns across the adult lifespan, with association systems showing greater age-related dedifferentiation than sensorimotor systems (Chan et al., 2014; Pedersen et al., 2021).

Sex differences in network-specific and global brain connectivity have been reported across the lifespan (Gong et al., 2011; Satterthwaite et al., 2015; Zhang et al., 2016). However, large connectivity studies that include older individuals are lacking. It, therefore, remains unclear whether robust sex differences in brain connectivity exist across the adult lifespan, especially in old age.

Cognitive performance varies greatly between individuals, and variability increases further with age (Tucker-Drob et al., 2019). Thus, recent studies have begun to explore whether the age-related reorganization of the brain is more efficient in certain individuals than others. A previous study reported that higher mean segregation of all systems (referred to as global segregation index) is related to better processing speed performance and higher crystallized intelligence in young adulthood (22 to 36 years) (Wang et al., 2021). Individuals with higher segregation of association systems were found to have better episodic memory performance, but did not differ in processing speed, verbal fluency, or verbal ability in two relatively small studies (n < 300) that included participants across a wide age range, from people in their twenties to eighties (Chan et al., 2014; Pedersen et al., 2021). In these studies, the level of sensorimotor systems segregation was neither related to episodic memory, nor to processing speed, verbal fluency, or verbal ability (Chan et al., 2014; Pedersen et al., 2021).

Cognitive tasks in which the sensorimotor systems might play a more important role are eye movement tasks. Eye movements are an alternative measure of a variety of cognitive processes, including attention, processing speed, working memory, learning and inhibition, and of motor performance (Hutton, 2008; Klein & Ettinger, 2019; Leigh & Zee, 2015). A previous study has shown that brain systems segregation is related to motor performance in bimanual coordination tasks (King et al., 2018). However, the relationship with oculomotor performance, which encompasses both cognitive and motor aspects, remains to be investigated.

Although the association between brain organization and cognition has been widely explored, the underlying causes and consequences of age-related dedifferentiation in brain organisation are still largely unknown. It has been assumed that dedifferentiation in brain organization is a compensatory mechanism to counteract age-related increases in the levels of neuropathology (Foo et al., 2021). Since neurofilament light chain (NfL) is a sensitive biomarker of neuroaxonal damage that can be measured in plasma (Khalil et al., 2018), NfL levels might be used to test the hypothesis that neuropathology is related to the age-related brain reorganization.

The aims of this project were twofold. First, we aimed to evaluate the effects of age and sex on brain systems segregation in a large population-based sample across a wide age range. Second, we sought to explore the underlying causes and consequences of the age-related brain (re)organization that we observed. Specifically, we tested in the overall sample and in different age strata whether the degree of brain systems segregation was related to cognitive performance in classical and eye movement tasks, and whether this relationship varied with the degree of neurodegeneration.

## 2 Methods

### 2.1 Participants

We based our study on the first 10,132 participants of the Rhineland Study, a community-based cohort study in Bonn, Germany. Study inclusion criteria are living in one of two geographically defined areas in Bonn and being at least 30 years or older. Participation is only possible upon invitation and there are no financial incentives. The only exclusion criterion is not having sufficient comprehension of the German language to provide written informed consent. Eligibility is irrespective of health status. The ethics committee of the Medical Faculty of the University of Bonn approved the study, which was carried out in accordance with the recommendations of the International Council for Harmonisation Good Clinical Practice standards (ICH-GCP). Participants underwent 8 hours of examinations, including a 1-hour cognitive examination. A flow chart in Supplementary Figure S1 shows the selection of the study population for this project. In short, imaging data of 6,695 participants were available. Of these 6,695 participants, 583 were excluded after quality control of the preprocessed fMRI data (details described in the next section) and 234 participants with more than 1-year interval between imaging acquisition and cognition assessment were also excluded. All but six participants had cognitive data from at least one examination. This left 6,455 participants for our analyses.

### 2.2 Imaging data acquisition and preprocessing

T1-weighted structural MRI (time of repetition (TR) = 2,560 ms, flip angle of 7°, field of view (FOV) = 256 x 256 mm, 0.8 mm isotropic) and 3D echo-planar imaging rs-fMRI scans (TR = 570 ms, TE = 30 ms, FOV = 216 mm, flip angle = 12°, 2.4 mm isotropic, number of volumes = 1053) were collected on one of the two identical 3T Siemens MAGNETOM Prisma MRI scanners with a 64-channel head coil (Koch et al., 2025). Participants were asked to keep their eyes open and fixate on a bright cross during the acquisition of resting-state fMRI.

We performed standard rs-fMRI preprocessing in SPM12 (Friston et al., 2007), including the following steps: (1) distortion correction, (2) motion correction, (3) spatial normalization, (4) smoothing (FWHM = 6 mm), and (5) temporal band-pass filtering. In addition, we used FMRIB’s ICA-based X-noisefier (FIX 1.06) to remove the non-neuronal noise components from the data (Griffanti et al., 2014; Salimi-Khorshidi et al., 2014). FIX was trained on a study-specific training dataset that was generated from 80 randomly selected subjects (equally distributed across age and sex strata) and hand-labeled by two independent raters (Griffanti et al., 2017). The mean portion of correctly labeled ‘signal’ and ‘noise’ components was 92.0% and 83.7% respectively. For each participant, we computed a first-level generalized linear model (GLM) using a discrete cosine basis set consisting of 93 functions that represent frequencies characteristic of resting-state dynamics of 0.0078–0.1 Hz, six head motion parameters, white matter and cerebral spinal fluid (CSF) signal intensities, and global signal drift. All processing and downstream functional connectivity (FC) extraction were performed in volumetric space.

Rs-fMRI data were acquired as the first sequence in our MRI protocol to minimize motion artifacts. As demonstrated in our prior work, participants exhibited significantly reduced head motion during rs-fMRI scanning compared to subsequent sequences (Pollak et al., 2023). Additionally, quality control was performed based on Power et al. (2017) to evaluate the quality of key preprocessing outputs and raw data. Scans with mean frame-wise displacement (FD) greater than 0.5 mm or a head displacement greater than 2 mm per frame were excluded. Scans with mean FD between 0.3 mm to 0.5 mm, with any frame with absolute head displacement >1.5 mm, or with less than 3 SD tSNR were visually inspected. We excluded scans showing either long excursion or sustained elevations in carpet plots (Supplementary Fig. S2). After our motion control scheme, residual motion effects were assessed with QC-FC plots (Ciric et al., 2017; Power et al., 2014) (Supplementary Fig. S3). Additional sensitivity analyses related to motion correction are shown in Supplementary Table S1.

### 2.3 Measures of brain functional connectivity

Regions of interest (ROIs) were defined using Gordon’s atlas, which includes 333 parcels assigned to twelve distinct functional systems (Gordon et al., 2016). The mean time series for each ROI were extracted and cross-correlated using Pearson’s product moment correlation, yielding a 333 x 333 FC matrix which was subsequently Fisher z-transformed. All negative correlations were set to zero and only positive values were retained.

We computed the system segregation index for global, association systems and sensorimotor systems using a previously established measurement taking the difference between mean within-system FC and mean between-system FC as a portion of mean within-system FC (Chan et al., 2014): $\frac{\text{FCwithin}-\text{FCbetween}}{\text{FCwithin}}$

FCwithin is the mean functional connectivity between ROIs within the same system, and FCbetween is the mean functional connectivity between ROIs of one system and all ROIs of the other systems.

To ensure that our results are not influenced by our choice of the 333-parcellation according to Gordon’s atlas, we additionally used the 400-parcellation scheme by Schaefer et al. (2018) as a sensitivity analysis.

### 2.4 Cognitive battery

We integrated ten performance measures from classical cognitive tasks and two measures from oculomotor cognitive tasks into the four domain scores processing speed, memory, executive function, and crystallized intelligence. For processing speed, we measured performance in a numbers-only trail-making test (TMT-A: time to completion) and prosaccade latency. Prosaccade latency represents the time needed to initiate a rapid eye movement, called saccade, toward a target that abruptly and randomly steps from the center of a computer screen to the left or right side (15 times per side). Memory was measured with digit span forward and backward, the Corsi block-tapping test (Corsi) forward and backward adapted from the PEBL battery (Mueller & Piper, 2014), and the Auditory Verbal Learning and Memory test (AVLT) (immediate recall: sum of correctly recalled nouns in the first five trials; delayed recall: number of correctly recalled words after a time delay of 20 to 30 minutes) (Boenniger et al., 2021). Executive function was measured with a number-and-letters switching trail-making test (TMT-B: time to completion), a 60 second categorical word fluency task (number of uniquely named animals), and antisaccade error rate. The antisaccade task has the same design as the prosaccade task but differs in the task instruction as participants are asked to look directly toward the mirror position of the target as soon as it steps from the centre of the computer screen to the left or right side. The antisaccade error rate is a measure of inhibitory control, representing the percentage of trials in which the initial saccade was made toward the target instead of the opposite direction. To measure crystallized intelligence, we used the 37-item Mehrfachwahl-Wortschatz-Intelligenztest (MWT-B), in which participants select an existing German word among four non-words in each of 37 trials (Lehrl, 2005). We calculated the domain scores based on averaged z-scores for the separate test scores in that domain. More details on how we calculated the domain scores can be found in a previous publication (Coors et al., 2022).

Apart from two eye movement measures that were included in the cognitive domain scores, we extracted additional measures from the prosaccade and antisaccade tasks to assess the associations with our three brain segregation indices. For both saccade tasks, we calculated mean latencies (time needed to initiate a saccade in ms), the two spatial accuracy measures amplitude gain and spatial error (both in %), and amplitude-adjusted and unadjusted peak velocities (in degree of visual angle/s). For the antisaccade task, we additionally calculated latency costs (in ms) and correction rate (the percentage of corrected antisaccade direction errors) in addition to the aforementioned antisaccade error rate (in %). Eye movements were recorded using video-based infrared oculography (EyeLink 1000 and EyeLink 1000 Plus; SR Research Ltd) at 1,000 Hz. A more detailed description of the oculomotor data acquisition and processing can be found in a recent publication (Coors et al., 2021).

### 2.5 Evaluation of neuropathology

We measured neurofilament light (NfL) protein level in plasma using the Simoa® NF-light Kit (103186) and an HD-1 or HD-X Analyzer (Quanterix, Billerica, USA). We adjusted the NfL values for the two covariates batch and rack position using a linear mixed-effects model (lmer function from the lme4 package version 1.1-26). In addition, we adjusted every model that included NfL for estimated glomerular filtration rate and blood volume, as these values are known to influence NfL levels (Akamine et al., 2020; Manouchehrinia et al., 2020). Blood volume was estimated using the Nadler equation (Sharma & Sharma, 2018), and estimated glomerular filtration rate was based on blood-derived cystatin C values (Inker et al., 2012). We excluded all NfL values that were more than five times the interquartile range above the third quartile of the corresponding 10-year age band (30–40 years, 40–50 years, 50–60 years, 60–70 years, 80+ years). Out of the 6,455 participants that we included in our main analyses, 3,480 (54.0%) had NfL values available. Selection of samples for NfL analysis was random, and therefore unbiased. Still, some participants refused to donate blood, so we checked if this might have introduced some bias. As shown in Table 1, participants with available NfL data were on average almost 1 year younger (mean age = 54.02 years, SD = 13.34) compared to the complete sample (mean age = 54.97, SD = 13.45) but they did not differ in terms of sex distribution, educational level, or level of visual acuity (Table 1). Educational level was classified according to the International Standard Classification of Education (ISCED) 2011. According to this classification system, low level of education refers to completed lower secondary education or less. Middle level of education refers to completed upper secondary education up to a completed bachelor’s degree or equivalent. Finally, high level of education refers to at least a completed master’s degree or equivalent. Visual acuity was measured with the automated refractometer (Ark-1s, Nidek Co., Tokyo, Japan) and classified based on the guidelines of the International Council of Ophthalmology.

**Table 1.** Demographic data of study population.

	Main sample	Participants with NfL available	p-value^1^
N	6,455	3,480	
Age, years (SD)	54.99 (13.35)	54.02 (13.34)	<0.001
Sex, N (%)			0.5
Women	3,040 (59%)	2,020 (58%)	
Men	2,130 (41%)	1,460 (42%)	
Education, N (%)			0.8
Low	87 (1.4%)	51 (1.5%)	
Middle	2,673 (42%)	1,434 (42%)	
High	3,580 (56%)	1,968 (57%)	
Best corrected visual acuity, N (%)			0.4
Low (< 0.32)	39 (0.6%)	22 (0.6%)	
Middle (0.32-0.63)	807 (13%)	404 (12%)	
High (≥ 0.8)	5,565 (87%)	3,027 (88%)	
Dementia, N (%)	4 (<0.1%)	3 (<0.1%)	0.7
NfL, pg/mL	8.81 (4.71)	8.81 (4.71)	0.14

^1^χ^2^ test was applied for comparison of sex, educational level, and best-corrected visual acuity; and a two-sample t-test was applied for comparison of age and plasma NfL level.

Abbreviations: NfL = neurofilament light.

Furthermore, we wished to investigate in more detail whether the association between global segregation and cognition depended on the level of neuropathology. In order to test our hypothesis that particularly individuals with low levels of neuropathology might benefit from high global segregation, we divided participants into high- (z-transformed plasma NfL level ≥ 0, adjusted NfL ≥ 28.09 pg/mL) and low- (z-transformed plasma NfL level < 0, adjusted NfL < 28.09 pg/mL) plasma NfL groups based on the values of the entire sample and then used the age strata as described below to test associations for the high and low NfL groups separately within each age stratum.

### 2.6 Statistical analyses

Statistical analyses were carried out in RStudio (version 1.3.959, R-base version 4.0.3). First, we quantified how global, sensorimotor, and association systems segregation differed across age and between men and women using one linear regression model per brain systems segregation index that included this index as a dependent variable and age and sex as independent variables. Next, we assessed whether sex differences in brain systems segregation vary with age by using a likelihood ratio test to test whether the inclusion of an age*sex interaction term improved the model.

To quantify associations between the global, the sensorimotor, and the association brain systems segregation indices and the four cognitive domain scores, we calculated a separate model for each combination between brain systems segregation index and cognitive domain score. This model included one brain systems segregation index as independent variable, one cognitive domain score as dependent variable, and age, age^2^, sex, and native language as additional variables to adjust for. Age and age^2^ were mean-centred to avoid multicollinearity. Next, we quantified the associations between the three brain systems segregation indices and the eye movement measures by calculating a separate model for each combination between brain systems segregation index and eye movement measure. In these models, we included one brain systems segregation index as independent variable and the prosaccade and antisaccade outcomes as dependent variable, additionally adjusting for age, age^2^, sex, and best-corrected visual acuity.

To evaluate whether associations between brain systems segregation indices and cognitive domain scores and oculomotor measures varied with age, we ran an additional model for each cognitive and oculomotor measure that included the two interaction terms “age*brain systems segregation index” and “age^2^*brain systems segregation index” in addition to the covariates mentioned above. We then evaluated with a likelihood ratio test whether the model with or without interaction terms better fitted the data. Since associations varied with age in many cases, we performed age-stratified analysis for the four age strata 30- to 45-year-olds, 46- to 55-year-olds, 56- to 65-year-olds, and 65+ -year-olds. We chose these age cut-offs to achieve approximately equal stratum sizes and because they may reflect important transition periods over the life course (MacDonald & Pike, 2021; Raz et al., 2005). Within each age stratum, we adjusted regression models for age, sex, and native language when cognition was the outcome, and for age, sex, and best-corrected visual acuity when oculomotor outcomes were analyzed.

We also evaluated whether the associations between the global brain systems segregation index and the cognitive domain scores and oculomotor measures differed between men and women by including a “sex*brain systems segregation index” interaction term in the basic models described in the first paragraph and evaluated with a likelihood ratio test the model fit.

We corrected all our analyses for multiple testing, so we corrected for N = 4 tests for the cognitive domain scores and N = 10 tests for the oculomotor measures. In the age-stratified analysis, we had 4 age strata and 4 cognitive domain scores, so we corrected for N = 16 tests. For the oculomotor measures, we had 4 age strata times 10 measures, so N = 40 tests. We applied the Benjamini and Hochberg (1995) false discovery rate (FDR) method to correct for multiple testing and considered an FDR-corrected p-value below 0.05 as statistically significant.

## 3 Results

### 3.1 Age-related differences in brain network organization are distinct between men and women

**Fig. 1.**
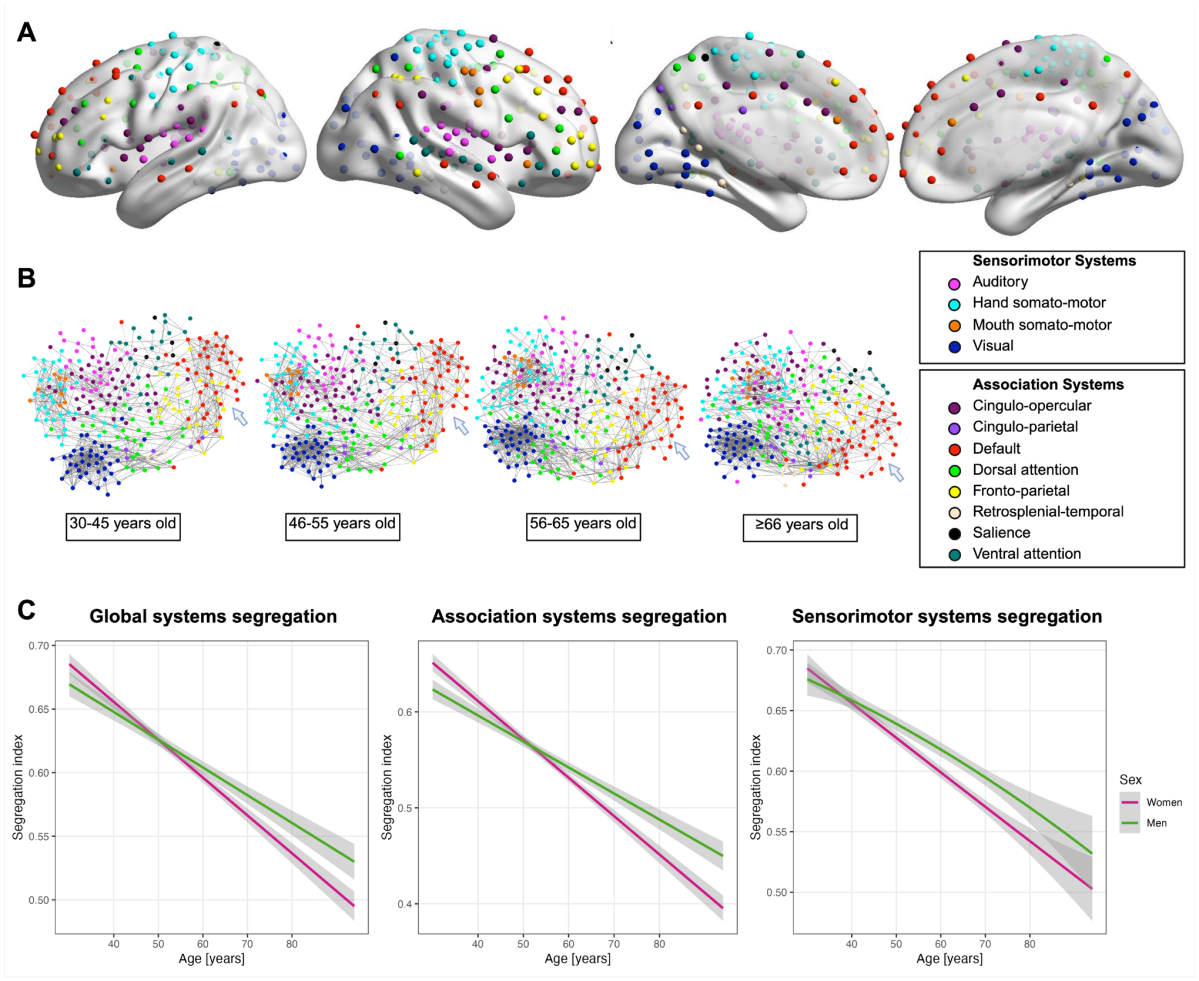
Age- and sex-related differences in brain systems segregation. (A) Brain functional network nodes are defined according to the brain atlas by Gordon et al. In total, there are N = 286 nodes belonging to 12 brain systems, which are shown in different colors. Nodes not assigned to any system are not shown (N = 47). (B) Spring-embedded graphs of the brain systems organization in younger (left), middle-aged (middle), and older adults (right). Graphs are derived from the mean functional connectivity of brain systems within each age group. Overall, the brain systems exhibited higher between systems functional connectivity and lower within systems functional connectivity in the group of older adults compared to the younger age groups (e.g., as highlighted for the default mode system by arrow). (C) Global segregation was negatively associated with age, with men showing higher global segregation than women after the age of about 50 years (left). Association system showed a linear association with age, with men having higher segregation than women after the age of about 50 years (middle). Sensorimotor system showed a non-linear association with age with men having higher segregation than women from about age of 40 years onwards (right).

Our analyses were based on 6,455 participants aged 30 to 95 years. Nearly 60% were women, and average educational level and best-corrected visual acuity were high (Table 1). To assess the status of brain systems organization, we divided the resting-state functional MRI data of the brain into 12 distinct functional systems (Fig. 1A) and determined functional connectivity within and functional connectivity between those brain systems. The effects of age and sex on brain systems segregation are shown in Figure 1B and C. Older age was associated with lower global brain systems segregation (ß = -0.298, 95% CI [-0.299, -0.297], p < 0.001), and across age, men showed higher global brain systems segregation than women (ß = 0.030, 95% CI [0.028, 0.031], p < 0.001). Furthermore, we found that the sex difference in global brain systems segregation was age-dependent, as the likelihood ratio test favored the model with an age*sex interaction term over the basic model (F-value = 10.91, p < 0.001). Figure 1C shows that sex differences in global brain systems segregation seemed to emerge only about the age of 50 years.

Since previous studies have shown that the association and sensorimotor systems have distinct patterns of age-related change, we also examined the associations between age and these two segregation indices. Overall, we found that age was negatively associated with segregation in both systems (Fig. 1C). For the association systems, we detected only a linear effect of age (ß = -0.352, 95%CI [-0.353, -0.351], p < 0.001), as the second-degree polynomial was not statistically significant. Similarly, the sensorimotor systems showed a linear trend across age with only significant first-polynomial (ß = -0.293, 95%CI [-0.294, -0.292], p < 0.001). In both sensorimotor and association systems, sex differences varied with age (sensorimotor: F-value = 9.449, p = 0.002, association: F-value = 23.389, p < 0.001). However, these systems showed slightly different patterns: in the association systems, segregation was higher in men from 50 years of age (ß = 0.027, 95% CI [0.025, 0.028], p < 0.001, Fig. 1C), while in the sensorimotor systems, segregation was higher in men from approximately 40 years of age compared to women (ß = 0.110, 95% CI [0.108, 0.111], p < 0.01, Fig. 1C).

### 3.2 Brain segregation is beneficial for cognition with the sensorimotor systems playing a crucial role for oculomotor performance

After adjusting for age, age^2^, sex, and native language, we found that a higher global systems segregation index was significantly associated with better memory performance before correction for multiple testing (ß = 0.018, 95% CI [0.004, 0.032], p = 0.014, p_FDR_ = 0.057), but not with any of the other domain scores (Table 2). Next, we related the sensorimotor and association systems segregation indices with cognitive domain scores to see if the association with cognition was system-specific. A higher degree of segregation in the association systems was significantly related to better memory performance (ß = 0.020, 95% CI [0.006, 0.035], p = 0.005, p_FDR_ = 0.041). This association between the segregation of the sensorimotor systems and memory was not significant after correction (ß = 0.017, 95% CI [0.003, 0.031], p = 0.016, p_FDR_ = 0.066). The two system segregation indices were not associated with the other cognitive domain scores (Table 2). When we performed a sensitivity analysis using segregation indices that were created based on the brain parcellation from Schaefer’s atlas, we found comparable results (see Supplementary Table S2).

**Table 2.** The associations between global, association, and sensorimotor system segregation indices and cognitive domain scores and oculomotor measures.

	Global systems		Association systems		Sensorimotor systems	
Cognitive domain	Beta [95% CI]	p-value	Beta [95% CI]	p-value	Beta [95% CI]	p-value
Crystallized intelligence	-0.011 [-0.038, 0.016]	0.415	-0.013 [-0.037, 0.011]	0.298	-0.010 [-0.034, 0.014]	0.429
Processing speed	0.006 [-0.010, 0.021]	0.473	0.006 [-0.010, 0.022]	0.419	0.006 [-0.009, 0.021]	0.446
Executive function	0.000 [-0.015, 0.016]	0.958	0.007 [-0.008, 0.023]	0.355	-0.002 [-0.017, 0.014]	0.836
Memory	0.018 [0.004, 0.032]	0.014*	0.020 [0.006, 0.035]	0.005**	0.016 [0.003, 0.031]	0.016*
Oculomotor measures						
Prosaccade latency (ms)	-0.004 [-0.027, 0.020]	0.762	-0.007 [-0.030, 0.017]	0.585	-0.007 [-0.030, 0.016]	0.546
Prosaccade amplitude gain (%)	-0.013 [-0.038, 0.013]	0.762	0.000 [-0.026, 0.026]	0.997	-0.003 [-0.029, 0.023]	0.837
Log of prosaccade spatial error (RMSE) (log %)	-0.013 [-0.038, 0.013]	0.333	-0.016 [-0.041, 0.010]	0.235	-0.005 [-0.031, 0.020]	0.692
Amplitude-adjusted peak prosaccade velocity	-0.009 [-0.035, 0.018]	0.510	-0.001 [-0.027, 0.026]	0.963	-0.010 [-0.036, 0.016]	0.470
Antisaccade latency (ms)	-0.024 [-0.048,0.001]	0.058	-0.026 [-0.050,-0.001]	0.042*	-0.030 [-0.056,-0.006]	0.014*
Antisaccade amplitude gain (%)	0.005 [-0.021, 0.032]	0.376	-0.002 [-0.028, 0.025]	0.910	0.019 [-0.008, 0.045]	0.171
Log of antisaccade spatial error (RMSE) (log %)	0.019 [-0.007, 0.046]	0.156	0.016 [-0.011, 0.043]	0.249	0.021 [-0.005, 0.048]	0.118
Amplitude-adjusted peak antisaccade velocity	-0.009 [-0.035, 0.018]	0.510	-0.001 [-0.027, 0.026]	0.971	-0.024 [-0.050, 0.003]	0.078
Antisaccade error rate (%)	-0.001 [-0.026, 0.024]	0.922	-0.013 [-0.038, 0.013]	0.321	0.009 [-0.016, 0.034]	0.470
Antisaccade costs (ms)	-0.028 [-0.054,-0.002]	0.035*	-0.028 [-0.054,-0.002]	0.038*	-0.033 [-0.060,-0.003]	0.012*

* indicates that an association was statistically significant at a significance level of p < 0.05, **indicates that an association was statistically significant at a significance level of p_FDR_ < 0.05 after correcting for multiple testing.

Abbreviations: CI = confidence interval; FDR = false discovery rate.

When we explored the association between the global segregation index and eye movement measures, we found that the global brain systems segregation index was weakly negatively associated with antisaccade costs (but not surviving correction). This suggests that individuals with higher levels of brain systems segregation needed less extra time to initiate a saccade in the opposite direction of a suddenly appearing target compared to initiating a saccade in the direction of a suddenly appearing target compared to individuals with lower levels of brain systems segregation (ß = -0.028, 95% CI [-0.054, -0.002], p = 0.035, p_FDR_ = 0.289) (Table 2). The global segregation index showed no associations with any other eye movement measures. When we examined the associations of sensorimotor and association systems segregation indices, we found that higher segregation of both segregation indices was weakly associated with lower antisaccade latency (association systems: ß = -0.026, 95% CI [-0.050, -0.001], p = 0.042, p_FDR_ = 0.208; sensorimotor systems: ß = -0.030, 95% CI [-0.056, -0.006], p = 0.014, p_FDR_ = 0.143) and lower antisaccade costs (association systems: ß = -0.028, 95% CI [-0.054, -0.002], p = 0.038, p_FDR_ = 0.208; sensorimotor systems: ß = -0.033, 95% CI [-0.060, -0.003], p = 0.012, p_FDR_ = 0.143; Table 2), but not surviving correction for multiple testing. The results using the segregation indices based on the brain parcellation scheme from Schaefer’s atlas show that higher values in the global, association, and sensorimotor system segregation indices were weakly associated with lower antisaccade latency and antisaccade costs—not surviving FDR-correction (Supplementary Table S2).

**Fig. 2.**
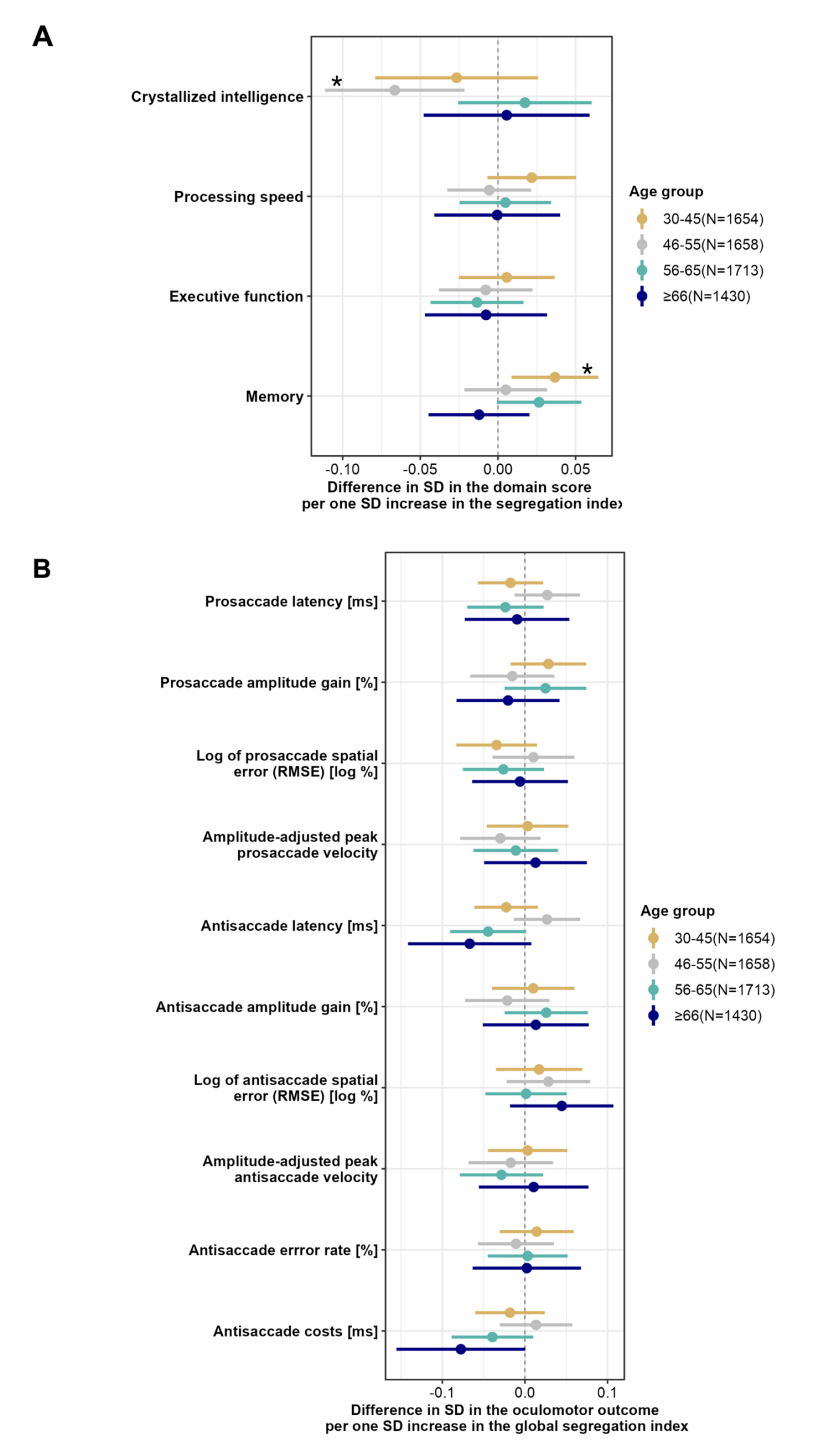
The association between global systems segregation and cognition with stratification for age. (A) The plot shows on the x-axis the differences in the cognitive domain scores per one SD increase in the global segregation index, separately for each age group. (B) The plot shows on the x-axis the differences in the oculomotor measures per one SD increase in the global segregation index, separately for each age group. *indicates that an association was statistically significant at a significance level of p < 0.05. Abbreviation: SD = standard deviation.

Subsequent age-stratified analyses revealed that both general and system-specific associations with memory performance were only present in the youngest age group of 30- to 45-year-olds (global systems segregation: ß = 0.037, 95%CI [0.009, 0.065], p = 0.010, p_FDR_ = 0.041, Fig. 2A). In the 46- to 55-year-olds, we found an association between global segregation index and crystallized intelligence. In this group, more segregated brain systems were associated with lower crystallized intelligence (ß = -0.066, 95%CI [-0.111, -0.022], p = 0.004, p_FDR_ = 0.015). However, no significant association was found between segregation index and eye movement measurements across all age groups.

When we repeated the age-stratified analyses using segregation indices based on Schaefer’s atlas, we could largely replicate the association between the global segregation index and crystallized intelligence in the 46–55 years age group (ß = -0.055, 95%CI [-0.099, -0.010], p = 0.016, p_FDR_ = 0.063). Although there was the same trend for the association between the global segregation index and memory performance in the youngest age group, it was not statistically significant (ß = 0.027, 95%CI [0.000, 0.055], p = 0.051, p_FDR_ = 0.206, Supplementary Fig. S4A).

### 3.3 Brain segregation mainly benefits participants with mild neuropathology

In light of the observation that the associations of segregation with cognition were driven by the younger age groups, it was important to test our hypothesis that high global brain systems segregation is only beneficial for cognitive performance in individuals with low levels of neurodegeneration. We hypothesized that associations between brain segregation and cognitive performance may be moderated by the level of neurodegeneration.

**Fig. 3.**
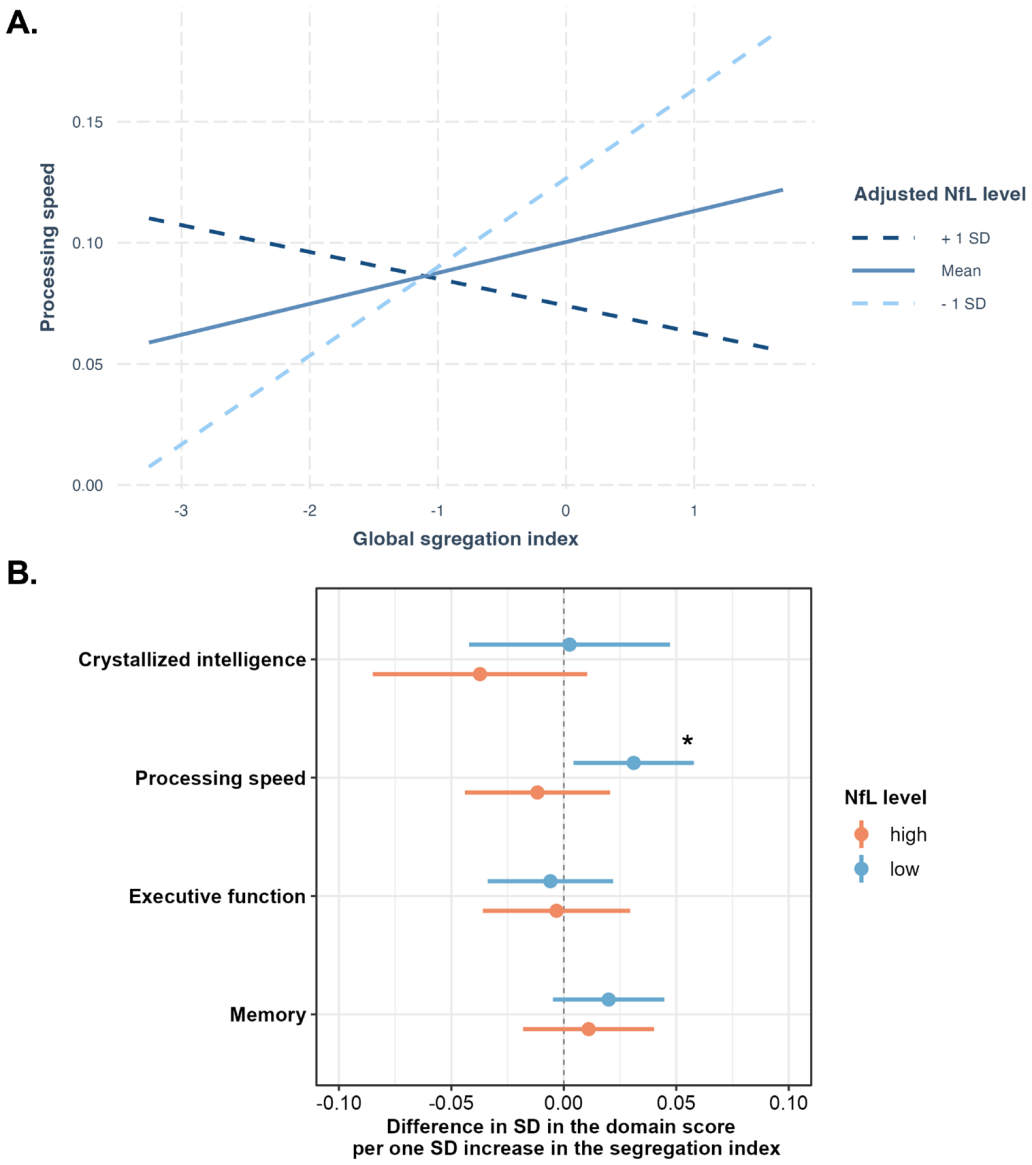
The association between global segregation index and cognition is affected by plasma NfL levels. (A) Interaction plot of global system segregation and plasma NfL level on processing speed. The plot shows that the association between global segregation index and processing speed is moderated by plasma NfL levels. The three lines represent the association between the global brain segregation index and processing speed in the group of participants with 1) dotted dark-blue line: one standard deviation above the mean plasma NfL level, 2) dotted light-blue line: one standard deviation below the mean plasma NfL level, and 3) blue line: mean plasma NfL level. The global segregation index and processing speed are both z-transformed. (B) The plot shows on the x-axis the differences in the cognitive domain scores per one SD increase in the global segregation index, stratified by high versus low plasma NfL levels. *indicates that an association was statistically significant at a significance level of p < 0.05. Abbreviation: SD = standard deviation; NfL = neurofilament light.

Therefore, we used plasma NfL as a marker of neurodegenerative pathology, with higher NfL levels indicating more neuroaxonal damage. We first examined whether the association between global brain segregation index and cognition was moderated by the NfL levels. Our analysis revealed a significant interaction effect between NfL and the global brain systems segregation index on processing speed (ß = -0.024, 95%CI [-0.043, -0.050], p = 0.013, p_FDR_ = 0.054, Fig. 3A), but no significant interaction effects were found for the eye movement measurements. Therefore, we stratified participants into high- (z-transformed plasma NfL level ≥ 0) and low- (z-transformed plasma NfL level < 0) plasma NfL groups. The mean NfL level value in the high NfL group was 11.5 pg/mL, compared to 6.7 pg/mL in the low NfL group. It is notable that the analysis was conducted in a subset of participants with available plasma NfL data (N = 3480), which limited the statistical power to detect associations in each group. Nevertheless, we found that with low NfL levels, higher global segregation was related to a higher processing speed domain score (processing speed: ß = 0.031, 95%CI [0.004, 0.058], p = 0.023, p_FDR_ = 0.182), whereas no association was found between global segregation and cognition in participants with high plasma NfL levels (Fig. 3B).

Our sensitivity analysis using the segregation indices based on Schaefer’s atlas showed broadly comparable results for the interaction between plasma NfL and global segregation on processing speed (ß = -0.025, 95%CI [-0.050, -0.001], p = 0.043, p_FDR_ = 0.172, Supplementary Fig. S5A). However, the association between the global segregation index and processing speed was not significant in the low plasma NfL group (ß = 0.028, 95%CI [-0.005, 0.060], p = 0.099, p_FDR_ = 0.395, Supplementary Fig. S5B).

## 4 Discussion

The aim of this project was to assess the relationships between age, sex, and brain systems segregation during resting-state and to investigate the links between brain (re)organization and cognitive performance as well as neuropathology as a possible underlying cause.

We were able to confirm previous findings that brain systems become less segregated with age. Also, our data confirmed that the association systems show a more accelerated decline in segregation over the adult lifespan compared to the sensorimotor systems (see also Betzel et al., 2014; Chan et al., 2014; Pedersen et al., 2021; Song et al., 2014). This implies that the association systems, in particular, benefit from multiple brain systems for processing complex information and that this is even more the case for older adults than for younger ones.

Concerning sex differences in brain systems segregation, a recent study on 22- to 36-year-olds reported stronger global and network-specific age-related dedifferentiation in women than men (Zhang et al., 2016). We confirmed this pattern but importantly observed that sex differences become stronger at a later age than previously reported, and earlier in the sensorimotor systems than in the association systems.

Our study also aimed at obtaining a better understanding of the reasons and consequences of age-related brain reorganization. Thus, we related the global, sensorimotor and association brain systems segregation indices to cognitive performance not only in our full sample, but also in different age and NfL level strata. Across the entire age range, we found that the global, the association, and the sensorimotor segregation indices were associated with memory performance, suggesting that brain functional specialization benefits memory processes, such as encoding, consolidation, and retrieval. This could be confirmed in our sensitivity analysis, in which we used brain segregation indices that were created based on the brain parcellation scheme of Schaefer et al. The association between the association systems segregation index and memory was strongest in our analysis and survived correction for multiple testing. In the sensitivity analysis, the relationships of both the association and the global segregation indices with memory survived correction for multiple testing. This suggests a degree of robustness to our findings and shows that especially segregation of the association systems seems to be beneficial for memory performance. A positive relationship between association systems segregation and memory performance has been reported by others (Chan et al., 2014). Our finding that the sensorimotor and global segregation index may also be linked to memory performance is novel (Chan et al., 2014; Pedersen et al., 2021). In our study, we comprehensively assessed memory for visual-spatial, numerical, and verbal material using multiple measures, which might explain why global brain organisation was also associated with memory performance before correction for multiple testing and why the sensorimotor systems were associated with memory in our sensitivity analysis. Our age-stratified analyses suggest that this association is driven by young adults (30 to 45 years).

Further, we found in the age-stratified analysis using both brain atlases, that higher global brain systems segregation was associated with *lower* crystallized intelligence in the 46–55 age group. In a previous study including individuals aged 22 to 36 years, higher global brain systems segregation had been found to benefit crystallized intelligence (Wang et al., 2021). This finding together with our finding suggests that the optimal brain organization for crystallized intelligence may change with age. It has been repeatedly shown that crystallized intelligence increases from young age to mid-adulthood, remains relatively stable through mid-to-late adulthood, and only declines in very late life, while performance in all other cognitive domains decreases earlier and substantially with age (Hayslip & Sterns, 1979). It has also been suggested that there are common neural mechanisms underlying the increase in crystallized intelligence and the decline in other cognitive domains (Tucker-Drob et al., 2019). Changes in the level of brain systems integration may be one such common mechanism underlying the age-related changes observed in the different cognitive domains. One possible reason why we did not find any association between brain systems segregation and crystallized intelligence in the two older age groups may be that the older age groups are more heterogeneous in terms of brain pathology. Also, we only found an association between brain systems segregation and memory in the youngest age group.

Thus, we tested whether associations between brain systems segregation and cognitive performance might be moderated by the level of neurodegeneration. Across the sample, we found that the association between the global segregation index and processing speed was moderated by NfL level. We visualized this moderation effect in Figure 3A, which shows that the direction of the association between global segregation index and processing speed reverses when the NfL level is high compared to when it is low. Subsequent stratification of our analyses by NfL level further showed that higher segregation was beneficial for processing speed only in those individuals with low levels of neurodegeneration. Findings from a previously published longitudinal study indicate that higher NfL levels relate to faster decline in functional connectivity within networks relevant for attention and memory. This suggests that neuropathology affects neuronal communication within specific networks, which may then contribute to cognitive decline (Dark et al., 2025). This together with our results suggests that individuals with higher levels of neuroaxonal loss may rely on brain systems integration as a compensatory mechanism to some extent to maintain high cognitive performance in the presence of neuropathology (Fornito et al., 2015). The increase in the dispersion of cognitive and oculomotor performance with age that has been observed in previous studies (Coors et al., 2021) suggests that not all individuals are equally good at compensating for these brain pathologies. A previous study reported that global systems segregation was positively associated with processing speed (Wang et al., 2021). However, the aforementioned study included participants aged between 22 and 36 years, who are likely to have lower levels of neurodegeneration than the participants from the Rhineland Study who are aged between 30 and 95 years. This discrepancy in age range may explain why an association between brain systems segregation and processing speed was not observed in the overall sample, but only in the NfL-stratified analysis.

It should also be noted that our data come from a population-based cohort and that we had excluded extreme NfL values (14 participants with more than five times the interquartile range above the third quartile of the corresponding 10-year age band [30–40 years, 40–50 years, 50–60 years, 60–70 years, 80+ years]) prior to our analyses. Thus, our comparison of high and low NfL groups does not imply that we are comparing healthy individuals with those suffering from neurodegenerative diseases. Instead, we are comparing two groups of individuals with a varying degree of normal age-related brain changes and some, but relatively minor, pathological brain changes. That absolute NfL levels were low is also evident when comparing the mean plasma NfL values in our high and low NfL groups (11.5 pg/mL and 6.7 pg/mL, respectively) to the mean plasma NfL levels that were reported in participants of the ADNI cohort that had dementia (45.9 pg/mL) or mild cognitive impairment (37.9 pg/mL)(Mattsson et al., 2019). Less than 0.1% of our participants who were included in the analysis had a diagnosis of dementia, which is a very low prevalence given the age range of 30 to 95 years. By focusing predominantly on healthy participants with varying degrees of neurodegeneration, we can better understand why we observed that brain specialiaation decreases across the adult lifespan. It should also be noted that most of our associations with cognitive and oculomotor measures were of small effect size and that some did not survive correction for multiple testing, which makes replication of our findings in an independent sample desirable.

The use of data from a relatively healthy population-based sample with a wide age range may also explain why we only observed interaction effects between NfL levels and brain segregation index on processing speed, but not on any other cognitive domain. According to Salthouse’s processing speed theory of age-related cognitive decline, processing speed is the cognitive domain that is affected first by aging (Salthouse, 1994). Thus, it may be the cognitive domain where, even in a relatively healthy sample, the association between brain organization and performance varies significantly depending on the degree of neurodegeneration. This explanation is further substantiated by a previous publication from our research group, which showed that higher NfL levels were associated with lower processing speed prior to correction for multiple testing, but not with any other cognitive domain score (Coors et al., 2025). This also suggests that the level of brain systems segregation relative to the degree of neurodegeneration may be a good indicator of how successfully someone is aging. Greater brain integration seems to be a way of compensating in the face of neurodegeneration, and in individuals where this brain restructuring is not occurring, this affects cognitive performance, particularly processing speed. The idea of brain integration being a compensatory mechanism also fits to the findings from a previous publication that showed that the rates of change in crystallized intelligence versus the other fluid abilities are strongly correlated (Tucker-Drob et al., 2022). However, brain integration is only one possible common mechanism, as strong interindividual differences in rates of change in crystallized intelligence and the other cognitive domains have been observed. While some individuals show a decline in fluid abilities along with a positive change in crystallized intelligence, others show a decline or an increase in all cognitive domains (Tucker-Drob et al., 2022).

Regarding the associations between brain systems segregation indices and eye movement measures, we found that all three segregation indices were initially associated with lower antisaccade costs in our main and sensitivity analyses. In addition, higher sensorimotor and association brain systems segregation indices were associated with lower antisaccade latency in the main analysis and all three segregation indices were associated with lower antisaccade latency in the sensitivity analysis. Although these seems to be a relatively robust findings as we could replicate it in the sensitivity analysis, none of the associations remained significant after correction for multiple testing, which indicates that associations were unreliable. Antisaccade latency depends on a variety of cognitive processes, including attention, speed of target detection, response-related decision-making, and response execution (Hutton, 2008). Since antisaccade costs are calculated by subtracting prosaccade latency from antisaccade latency, it is closely related to the findings for antisaccade latency. Thus, our findings suggest that brain systems segregation may benefit fast antisaccade initiation.

Overall, our results indicate that the optimal type of brain organization depends on the degree of brain pathology. Thus, brain reorganization in adulthood might be a compensatory mechanism to deal with increasing neuropathology resulting from normal aging and diseases.

## Supplementary Material

Supplementary Material

## Data Availability

The datasets for this manuscript are not publicly available because of data protection regulations. Access to data can, however, be provided to scientists in accordance with the Rhineland Study’s Data Use and Access Policy. Requests to access the datasets should be directed to Dr Monique Breteler, RS-DUAC@dzne.de.
