# Supplementary material for: Neuropathology determines whether brain systems segregation benefits cognitive performance
Source: Imaging Neurosci (Camb). 2025 Sep 9;3:IMAG.a.138. doi: 10.1162/IMAG.a.138 (PMC12421694; doi:10.1162/IMAG.a.138)
Supplement: Supplementary Material [file IMAG.a.138_supp.pdf]

Supporting Information for

Neuropathology determines whether brain systems segregation benefits cognitive performance

Annabell Coors[a,b,c,1], Weiyi Zeng[a,1], Ulrich Ettinger[d], and Monique M.B.Breteler[a,e,2]

[a] Population Health Sciences, German Center for Neurodegenerative Diseases (DZNE), Bonn, Germany;
[b] Cognitive Neuroscience Division, Department of Neurology, Columbia University Vagelos College of Physicians and Surgeons, New York, USA;
[c] Department of Psychiatry, Psychotherapy and Psychosomatic Medicine, University Medical Center Halle, Halle (Saale), Germany;
[d] Department of Psychology, University of Bonn, Germany;
[e] Institute for Medical Biometry, Informatics and Epidemiology (IMBIE), Faculty of Medicine, University of Bonn, Germany
[1] A.C. and W.Z. contributed equally to this work.
[2] To whom correspondence should be addressed. E-mail: monique.breteler@dzne.de

This SI Appendix includes:

1. **Supplementary figures:**
   1.1 Supplementary Figure S1: Selection of study population
   1.2 Supplementary Figure S2: Illustration of de-noising strategies
   1.3 Supplementary Figure S3: Residual QC-FC correlations after de-noising
   1.4 Supplementary Figure S4: The association between global systems segregation index and cognition based on Schaefer's atlas.
   1.5 Supplementary Figure S5: The association between global systems segregation index and cognition (based on Schaefer's atlas) is affected by plasma NfL levels.

2. **Supplementary table:**
   2.1 Supplementary table S1: Inclusion of quality assurance metrics does not substantially alter the observed relationships
   2.2 Supplementary table S2: The associations between global, association, and sensorimotor systems segregation indices and cognitive domain scores and oculomotor measures using the brain parcellation scheme by Schaefer et al..

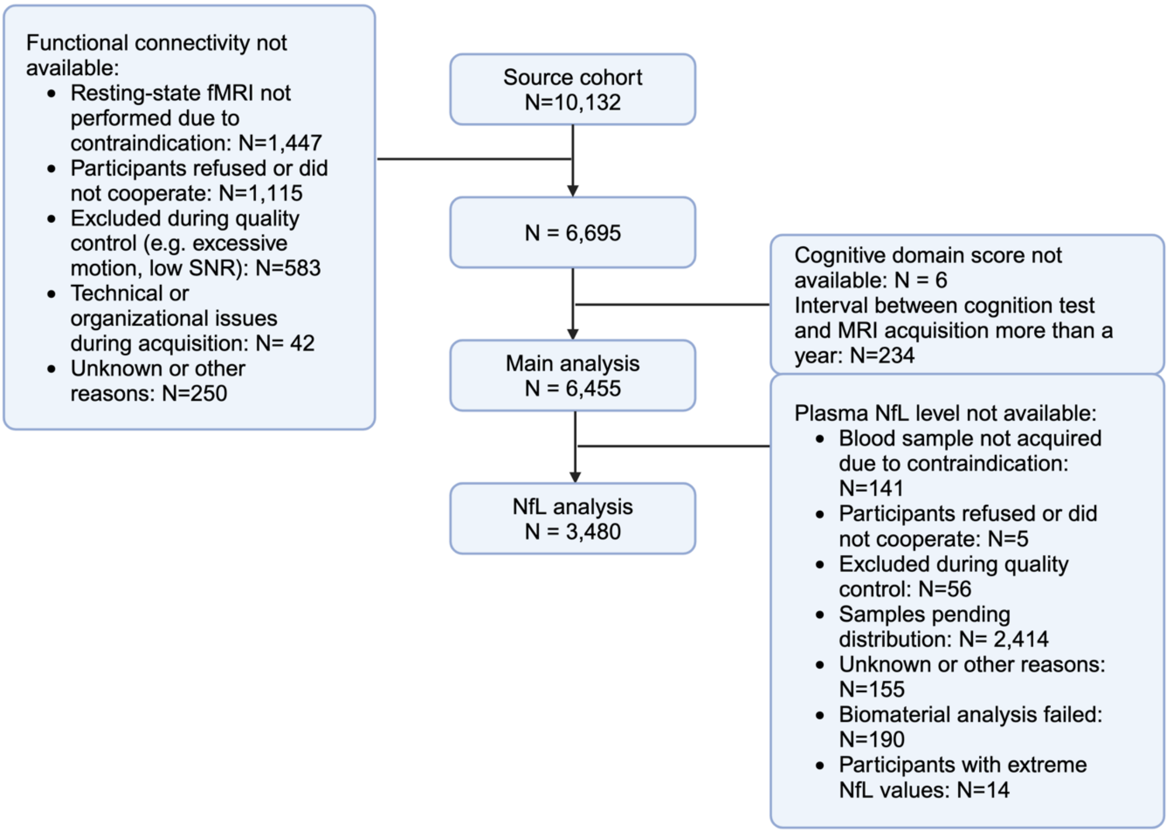

Supplementary Figure S1: **Selection of study sample.** The source cohort consisted of the first 10,132 participants of the Rhineland Study.
Abbreviations: NfL = neurofilament light, fMRI = functional Magnetic Resonance Imaging, SNR = signal-to-noise ratio.

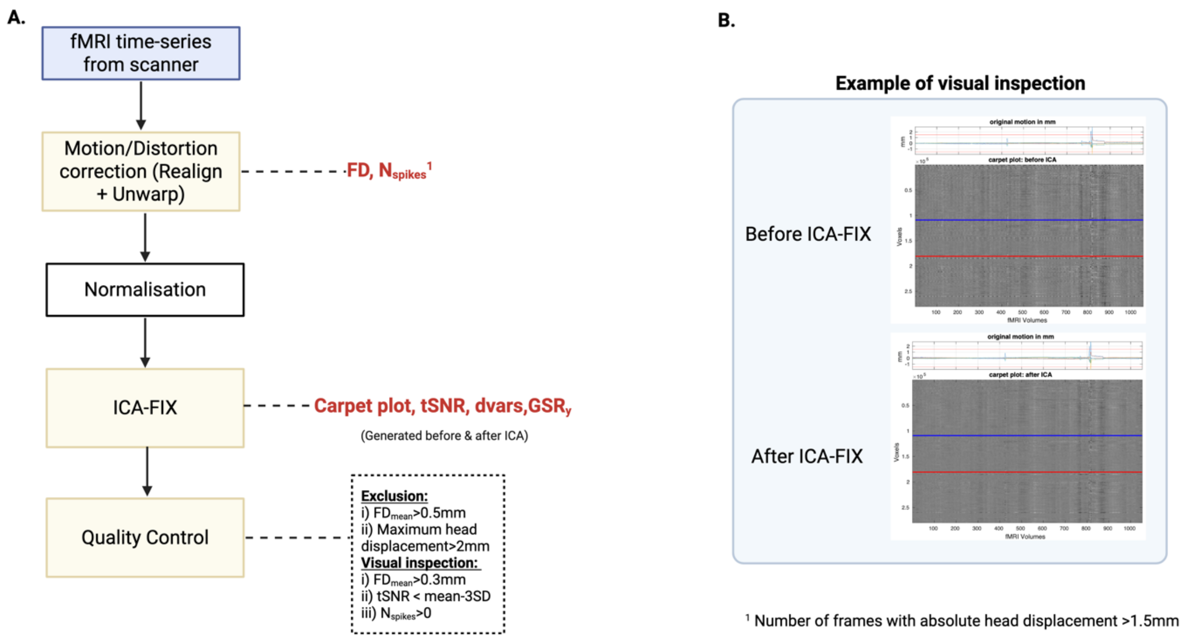

Supplementary Figure S2: Illustration of de-noising strategies.

A. The boxes depict the general BOLD processing strategy. Yellow boxes depict the main strategies related to de-noising.

B. An example of visual inspection. Carpet plots show an excluded participant due to spikes of head displacement over 2mm.

[1] Number of frames with absolute head displacement >1.5mm

Abbreviations: FD = framewise displacement, tSNR = temporal signal-to-noise ratio, GSR = ghost-to-noise ratio

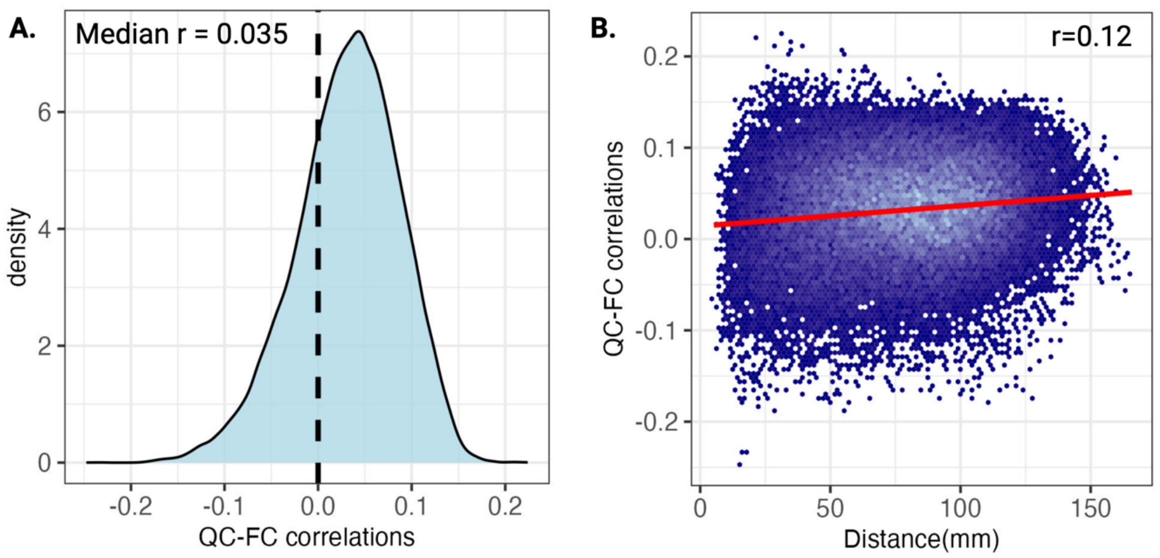

Supplementary figure S3: Residual QC-FC correlations after de-noising.

A. Distribution of all edgewise QC-FC correlations after de-noising strategy, in 333-node network as defined by Gordon et al. (2016).

B. Density plot indicating the relationship between the Euclidean distance separating each pair of nodes (x-axis) and the QC-FC correlation of the edge connecting those nodes (y-axis). The overall trend line from which distance-dependence is computed is indicated in red. R value is calculated using Pearson's correlation.

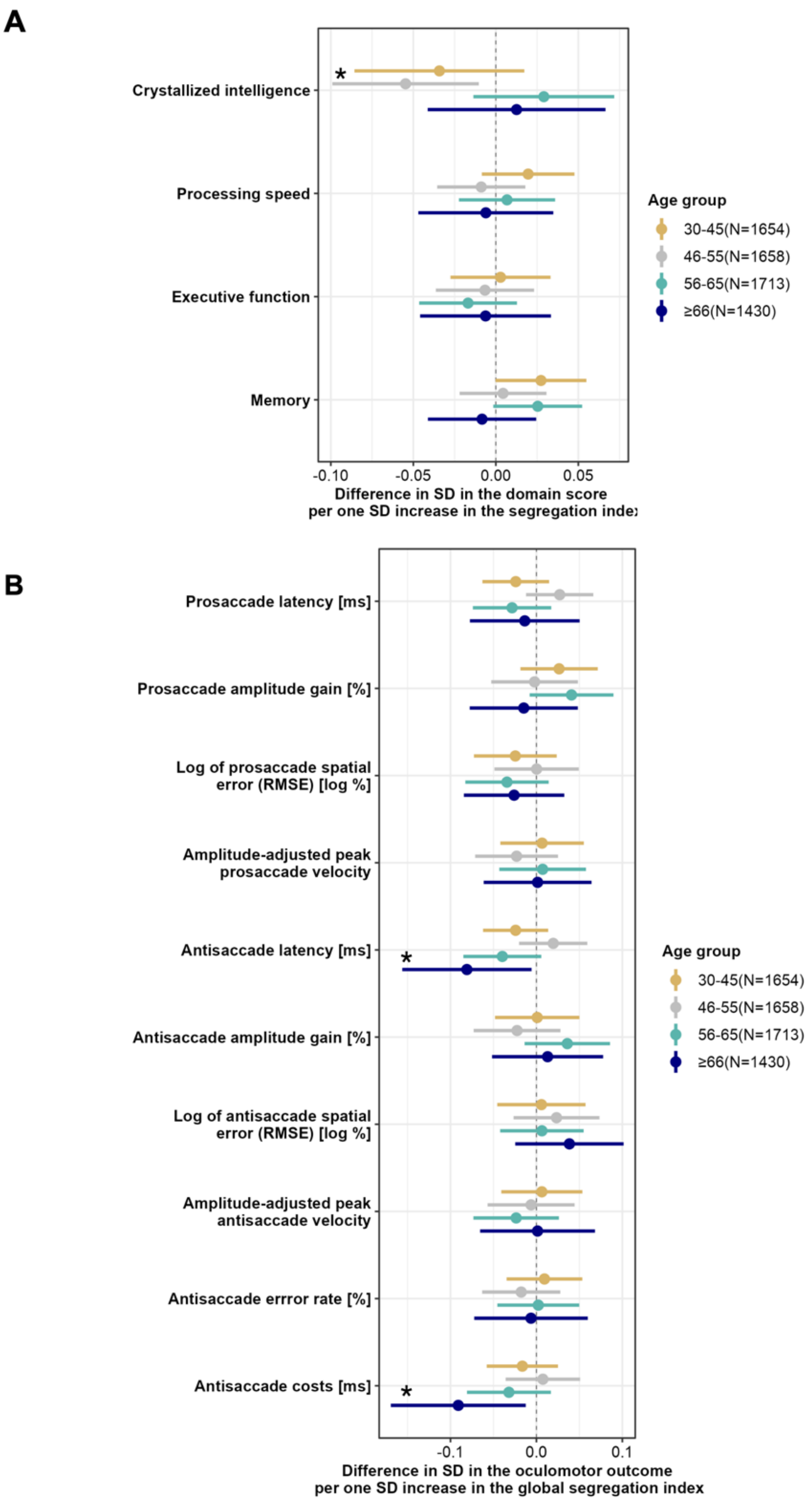

Figure S4. The association between global systems segregation index and cognition based on Schaefer's atlas

A. The forest plot shows on the x-axis the differences in the cognitive domain scores per one SD increase in the global systems segregation index, separately for each age group.

B. The forest plot shows on the x-axis the differences in the oculomotor measures per one SD increase in the global systems segregation index, separately for each age group.

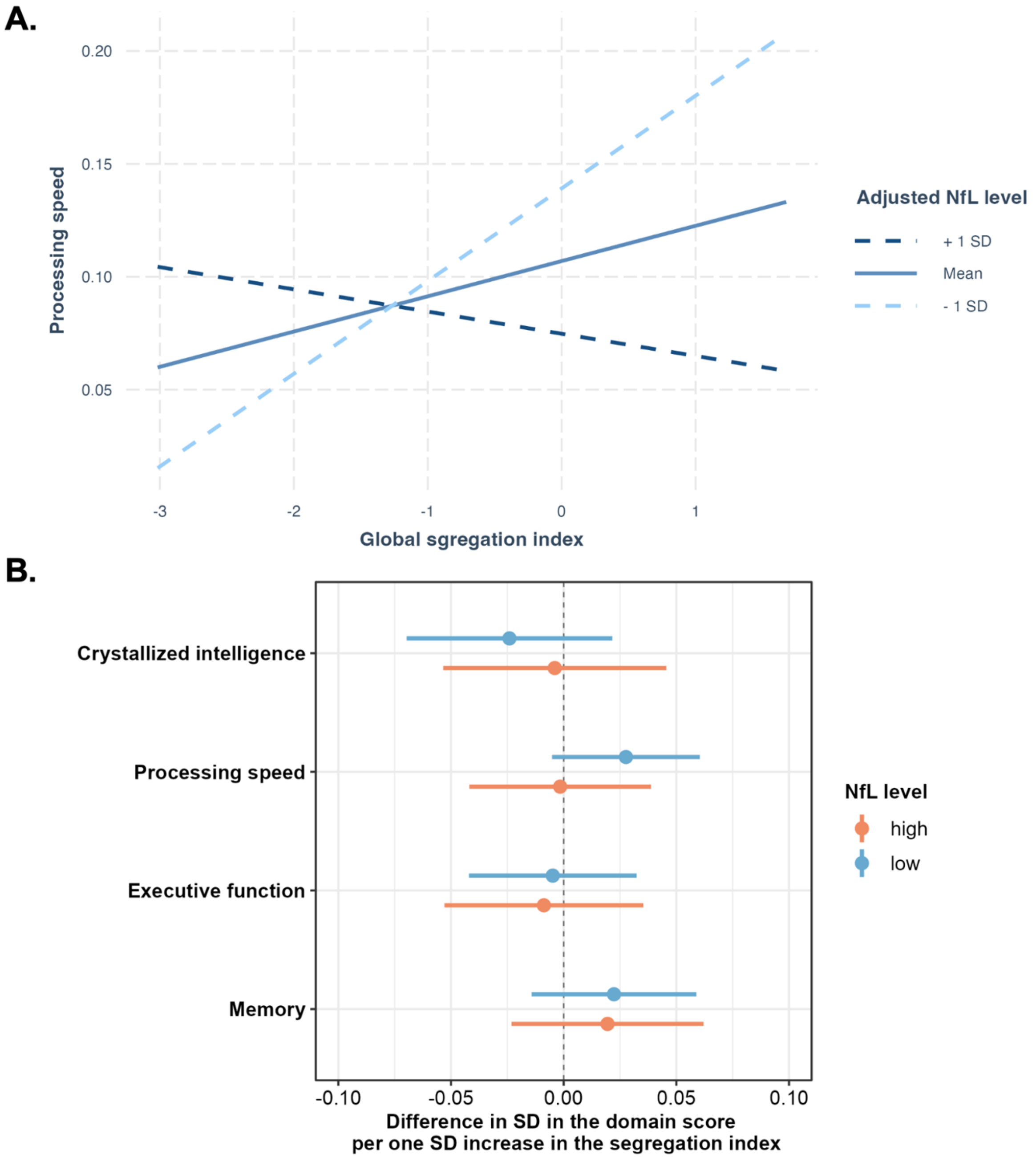

Figure S5. The association between global systems segregation index (based on Schaefer's atlas) and cognition is affected by plasma NfL levels.

A. Interaction plot of global systems segregation index and plasma NfL level on processing speed. The plot shows that the association between global systems segregation index on processing speed is moderated by plasma NfL level. The lines represent the association in the group of participants with one standard deviation above and below the mean plasma NfL level and the association in the group of participants with mean plasma NfL level. The global systems segregation index and processing speed are z-transformed.

B. The forest plot shows on the x-axis the differences in the cognitive domain scores per one SD increase in the global segregation index stratified by different plasma NfL levels.

Abbreviations: SD = standard deviation; NfL = neurofilament light.

Table S1. Inclusion of quality assurance metrics does not substantially alter the observed relationships

Regression Models	Without FD&G/S-ratio		With FD&G/S-ratio	
	**standardized *β***	***p***	**standardized *β***	***p***
**Global systems segregation vs. Age[1]**	-0.298	<0.001	-0.260	<0.001
**Association system segregation vs. Age[1]**	-0.352	<0.001	-0.297	<0.001
**Sensorimotor system segregation vs. Age[1]**	-0.293	<0.001	-0.257	<0.001
**Memory vs. Global system segregation[2]**	0.018	0.014	0.014	0.047
**Memory vs. Association system segregation[2]**	0.020	0.005	0.016	0.027
**Memory vs. Sensorimotor system segregation[2]**	0.016	0.016	0.014	0.043
**Antisaccade cost vs. Global system segregation[3]**	-0.028	0.035	-0.026	0.051
**Antisaccade cost vs. Association system segregation[3]**	-0.028	0.038	-0.026	0.060
**Antisaccade cost vs. Sensorimotor system segregation[3]**	-0.033	0.012	-0.032	0.017

[1] adjusted for sex
[2] adjusted for age, age^2, sex, native language (German versus other language)
[3] adjusted for age, age^2, sex, visual acuity
Abbreviations: FD = mean frame-wise displacement; G/S-ratio = ghost-to-signal ratio

Table S2. The associations between global, association, and sensorimotor systems segregation indices and cognitive domain scores and oculomotor measures using the brain parcellation scheme by Schaefer.

	Global systems		Association systems		Sensorimotor systems	
**Cognitive domain**	**Beta [95% CI]**	**p-value**	**Beta [95% CI]**	**p-value**	**Beta [95% CI]**	**p-value**
**Crystallized intelligence**	-0.011 [-0.034, 0.013]	0.383	-0.009 [-0.033, 0.015]	0.466	-0.004 [-0.028, 0.020]	0.752
**Processing speed**	0.004 [-0.011, 0.019]	0.593	0.005 [-0.011, 0.020]	0.549	0.006 [-0.009, 0.021]	0.443
**Executive function**	0.001 [-0.014, 0.017]	0.880	0.005 [-0.010, 0.020]	0.515	0.004 [-0.011, 0.020]	0.582
**Memory**	0.017 [0.003, 0.031]	0.019*	0.018 [0.004, 0.032]	0.012**	0.019 [0.005, 0.034]	0.006**
**Oculomotor measures**						
**Prosaccasde latency (ms)**	-0.007 [-0.030, 0.016]	0.550	-0.005 [-0.028, 0.018]	0.657	-0.007 [-0.031, 0.016]	0.528
**Prosaccasde amplitude gain (%)**	0.010 [-0.016, 0.035]	0.446	0.013 [-0.013, 0.038]	0.326	0.007 [-0.018, 0.033]	0.572
**Log of prosaccade spatial error (RMSE) (log %)**	-0.019 [-0.044, 0.006]	0.139	-0.021 [-0.047, 0.004]	0.096	-0.016 [-0.042, 0.009]	0.211
**Amplitude-adjusted peak prosaccade velocity**	-0.001 [-0.027, 0.025]	0.956	0.002 [-0.024, 0.028]	0.896	-0.003 [-0.029, 0.023]	0.818
**Antisaccade latency (ms)**	-0.027 [-0.051, -0.003]	0.027*	-0.024 [-0.049, 0.000]	0.047*	-0.033 [-0.057, -0.008]	0.008*
**Antisaccade amplitude gain (%)**	0.005 [-0.021, 0.032]	0.692	0.002 [-0.024, 0.028]	0.874	0.012 [-0.014, 0.039]	0.359
**Log of antisaccade spatial error (RMSE) (log %)**	0.015 [-0.011, 0.041]	0.269	0.015 [-0.011, 0.041]	0.264	0.014 [-0.012, 0.040]	0.298
**Amplitude-adjusted peak antisaccade velocity**	-0.005 [-0.032, 0.021]	0.668	0.000 [-0.027, 0.026]	0.983	-0.015 [-0.042, 0.011]	0.256
**Antisaccade error rate (%)**	-0.006 [-0.031, 0.019]	0.614	-0.014 [-0.039, 0.011]	0.259	-0.004 [-0.029, 0.021]	0.753
**Antisaccade costs (ms)**	-0.030 [-0.056, -0.004]	0.024*	-0.027 [-0.053, -0.002]	0.038*	-0.036 [-0.062, -0.010]	0.007*

*indicates that an association was statistically significant at a significance level of $p<0.05$, **indicates that an association was statistically significant at a significance level of $p_{FDR}<0.05$ after correcting for multiple testing
Abbreviations: CI = confidence interval; FDR = false discovery rate.
